# Rescue technique using natural orifice transluminal endoscopic surgery for stent migration into the peritoneal cavity during endoscopic ultrasound-guided biliary drainage

**DOI:** 10.1055/a-2744-8566

**Published:** 2025-12-17

**Authors:** Wenjuan Ding, Shan-Shan Hu, Xiang-Rong Zhou, Da-Hai Xu, Wei-Hui Liu

**Affiliations:** 1 546231 Department of Gastroenterology, Jianyang People’s Hospital, Jianyang, China; 2 89669 Department of Gastroenterology and Hepatology, Sichuan Provincial Peopleʼs Hospital, School of Medicine, University of Electronic Science and Technology of China, Chengdu, China

Endoscopic ultrasound-guided biliary drainage (EUS-BD) is a critical salvage intervention for malignant biliary obstruction after failed endoscopic retrograde cholangiopancreatography (ERCP 1 2 3 ). Stent migration into the abdominal cavity represents a rare but life-threatening complication 4 5 . We describe a novel application of natural orifice transluminal endoscopic surgery (NOTES) for urgent retrieval following stent displacement during EUS-BD.

A 72-year-old patient with unresectable cholangiocarcinoma and recurrent cholangitis post-PTCD was referred for internal drainage. After failed ERCP due to duodenal papilla tumor involvement, EUS-BD was performed but complicated by distal stent migration into the peritoneal cavity ( Fig. 1 **a** ). To achieve immediate retrieval, we performed an endoscopic NOTES procedure. The gastric wall was incrementally incised through the original puncture site ( Fig. 1 **b** ). Initial fluoroscopy-guided attempts to grasp the stent failed due to misalignment in anatomical planes ( Fig. 1 **c** ). Consequently, the gastric incision was extended toward the stent under fluoroscopic guidance ( Fig. 1 **d** ). Under fluoroscopic guidance, the omentum was incised toward the liver ( Fig. 1 **e** ). Upon visualization of the liver, the inferior border was visually inspected and traced to locate the stent. The site where the stent protruded through the hepatic surface was identified upon encountering the portion of the liver with adherent blood clots ( Fig. 1 **f** ). The stent was promptly secured with forceps and extracted ( Fig. 1 **g** ). After confirming the absence of active bleeding or peritoneal fluid, the gastric incision was sequentially closed using endoscopic clips in a linear configuration ( Fig. 1 **h** ). The patient maintained hemodynamic stability throughout the procedure with no perioperative complications, including hemorrhage, infectious sequelae, or delayed adverse events.

**Fig. 1:**
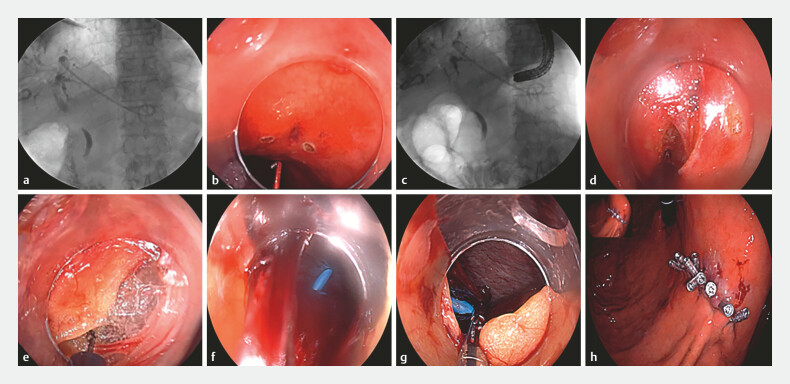
A schematic of the NOTES as salvage therapy for stent migration in EUS-BD. **a** Postprocedural imaging showed stent migration, with proximal retention in the intrahepatic biliary system and distal displacement into the peritoneal cavity through the hepatogastric space. **b** The gastric wall layers were sequentially divided through the initial puncture tract. **c** Despite apparent fluoroscopic alignment, initial attempts at direct stent capture with grasping forceps failed due to spatial discordance between the forceps and stent planes. **d** The gastric incision was extended toward the stent under fluoroscopic guidance. **e** Under fluoroscopic guidance, the omentum was incised toward the liver. **f** We identified the site of stent protrusion through the hepatic surface upon encountering the liver segment with adherent blood clots. **g** The stent was grasped under direct vision and retrieved immediately. **h** The gastric defect was closed endoscopically using through-the-scope clips with complete mucosal apposition.

This case demonstrates the feasibility of NOTES for the urgent management of migrated biliary stents, circumventing the morbidity of laparotomy. The NOTES technique combines endoscopic precision with minimally invasive principles, enabling rapid foreign body extraction while mitigating risks associated with prolonged abdominal cavity stent retention. This method is a rapid, effective and less invasive endoscopic treatment for stent detachment ( Video 1 ).

A rescue technique using NOTES for stent migration into the peritoneal cavity during EUS-guided biliary drainage.Video 1

Endoscopy_UCTN_Code_CPL_1AL_2AD

## Notes

Endoscopy E-Videos https://eref.thieme.de/e-videos
*E-Videos* is an open access online section of the journal *Endoscopy* , reporting on interesting cases and new techniques in gastroenterological endoscopy. All papers include a high-quality video and are published with a Creative Commons CC-BY license. Endoscopy E-Videos qualify for HINARI discounts and waivers and eligibility is automatically checked during the submission process. We grant 100% waivers to articles whose corresponding authors are based in Group A countries and 50% waivers to those who are based in Group B countries as classified by Research4Life (see: https://www.research4life.org/access/eligibility/ ). This section has its own submission website at https://mc.manuscriptcentral.com/e-videos .
